# Unsafe clinical practices as perceived by final year baccalaureate nursing students: Q methodology

**DOI:** 10.1186/1472-6955-11-26

**Published:** 2012-11-26

**Authors:** Laura A Killam, Phyllis Montgomery, June M Raymond, Sharolyn Mossey, Katherine E Timmermans, Janet Binette

**Affiliations:** 1 School of Health Sciences and Emergency Services, Cambrian College, 1400 Barrydowne Road, Sudbury, Ontario, Canada; 2 School of Nursing, Laurentian University, 935 Ramsey Lake Road, Sudbury, ON, Canada

**Keywords:** Nursing education, Q-Methodology, Safety, Clinical learning, Student perspectives

## Abstract

**Background:**

Nursing education necessitates vigilance for clinical safety, a daunting challenge given the complex interchanges between students, patients and educators. As active learners, students offer a subjective understanding concerning safety in the practice milieu that merits further study. This study describes the viewpoints of senior undergraduate nursing students about compromised safety in the clinical learning environment.

**Methods:**

Q methodology was used to systematically elicit multiple viewpoints about unsafe clinical learning from the perspective of senior students enrolled in a baccalaureate nursing program offered at multiple sites in Ontario, Canada. Across two program sites, 59 fourth year students sorted 43 theoretical statement cards, descriptive of unsafe clinical practice. Q-analysis identified similarities and differences among participant viewpoints yielding discrete and consensus perspectives.

**Results:**

A total of six discrete viewpoints and two consensus perspectives were identified. The discrete viewpoints at one site were *Endorsement of Uncritical Knowledge Transfer, Non-student Centered Program* and *Overt Patterns of Unsatisfactory Clinical Performance.* In addition, a consensus perspective, labelled *Contravening Practices* was identified as responsible for compromised clinical safety at this site. At the other site, the discrete viewpoints were *Premature and Inappropriate Clinical Progression, Non-patient Centered Practice* and *Negating Purposeful Interactions for Experiential Learning.* There was consensus that *Eroding Conventions* compromised clinical safety from the perspective of students at this second site.

**Conclusions:**

Senior nursing students perceive that deficits in knowledge, patient-centered practice, professional morality and authenticity threaten safety in the clinical learning environment. In an effort to eradicate compromised safety associated with learning in the clinical milieu, students and educators must embody the ontological, epistemological and praxis fundamentals of nursing.

## Background

Patient safety is broadly understood as the commitment to preventing healthcare errors or harm [1,2]. From a benevolent orientation, the mitigation of adverse health events is optimized through vigilance and commitment to evidence informed practice. Patient safety is recognized as a transglobal mandate central to nursing care across all sectors and settings [3,4]. Beyond a mandate, the Canadian Nurses Association characterizes the commitment to patient safety as an ethical obligation. For nursing students, as novices within the profession, it is imperative that patient safety is internalized as both a central humanistic nursing value and a fundamental patient right [5].

An indicator of compromised patient safety is the occurrence of adverse health events. Globally, the reported incidence of such events ranges between 4% and 17% influenced by diverse contextual variables [6-8]. Researchers suggest that up to half of all reported adverse events which compromised patient safety are preventable [9,10]. This healthcare tragedy requires individuals, practice organizations, educational institutions, and systems to commit to actualizing a shared evidence-informed culture of patient safety by preventing, identifying, reporting and analyzing errors [11,12].

Research on patient safety has predominantly focused on acute care errors [6,13] and more recently, those particular to primary care practices [8]. Given that substantial student learning experiences occur within acute care settings, educators are challenged to proactively engage in the development of patient safety curricula. The Canadian accrediting body for baccalaureate schools of nursing, advocates that the responsibility for student education must be shared by practice and academic institutions [14]. At present, there is no national strategy that prescribes a unified approach to address patient safety within nursing curricula. There are no standards regarding the inclusion of specific courses on safety, or number of hours for teaching safety within classroom, laboratory or clinical settings. Further, there is no database to capture the scope and depth of safety related initiatives across individual baccalaureate programs. Educators in partnership with stakeholders autonomously plan, implement and evaluate initiatives for clinical safety. Localized curricular initiatives that address safety within the clinical setting are beginning to emerge in the literature [15,16].

Within clinical education, identification and management of an unsafe student remains a challenging and time-consuming process, requiring faculty support and guidance [17]. Although characteristics of unsafe students identified within the literature help to identify potentially problematic situations, clarity of clinical expectations and an understanding of what constitutes unsafe practice is needed. These guidelines would facilitate consistent identification of potential threats to patient safety and student remediation [17,18]. The Canadian Association of Schools of Nursing advocates for a systems approach to safety in education informed by student input [14]. Only one study was found examining student perceptions of unsafe clinical learning situations [19]. Additional research is warranted to further develop evidence-informed safety focused nursing education programs. This study describes undergraduate nursing students’ emic understanding of unsafe practices and contexts that have the potential to cause harm. The specific topic of inquiry involved an identification of when nursing students perceived clinical practice to be most unsafe.

### Literature review

Patient safety is often viewed as a responsibility shared by all participants in the health care system [20]. While individual practitioners are indeed accountable for the quality of their work, patient safety is optimized by focusing on system processes rather than exclusively on individual performance [3,12,14,21]. Partners within a system approach include regulatory bodies, educational institutions, diverse practice organizations, and the individuals within these structures that provide and receive services. In this network, patient safety education must be integrated into formal health care programs across all disciplines and levels of learning [20]. Although undergraduate nursing education includes components that assist in the fulfillment of a patient safety mandate, some have argued for the expansion and refinement of curricular content [22,23]. Further, it has been suggested that the safety focus be made explicit rather than implicit within undergraduate nursing programs [24]. It has been recommended that comprehensive patient safety knowledge requires focused study in areas such as infection control, environmental hazards and patient response to high risk situations [25]. A barrier to achieving an efficient curricular transformation is the dearth of evidence to guide educators in the development of dedicated safety education [26-29].

A contemporary initiative involving multiple undergraduate nursing programs in the United States is the Quality and Safety Education for Nurses (QSEN)[30,31]. It focuses on the enhancement of nursing curricula to support student achievement of quality and safety competencies. The six QSEN competencies, integrated throughout the curricula, include patient-centered care, teamwork and collaboration, evidence-based practice, quality improvement, safety, and informatics. Overall, this project is intended to provide nurses with safety knowledge, skills and attitude in order to improve the quality and safety of the health care systems in which they work. Sullivan and associates [32] endorsed the development of quality and safety competencies during undergraduate nursing education as important for safe professional practice.

Practitioners are confronted with an increasingly complex health care environment. This complexity is defined by an interaction among variables such as economics, multi-level human resources, constant change, unpredictability, communication and relational challenges, advancing technology, and multifaceted health needs [12,20,27]. Such a practice context requires safety conscious health care providers to sustain a culture of safety. Richardson and Storr’s review of nursing literature suggest that leadership, collaboration, and empowerment as demonstrated are fundamental to safeguarding patients [1]. These hallmark elements were also identified in a dimensional concept analysis on patient safety culture [33]. Further, it has been suggested that sound judgment mediates the complexity of the practice context and thereby minimizes negative health care outcomes for patients [1,34].

Approximately one half of new nurses with less than one year of experience and involved in adverse patient events identified that their formal educational preparation as a causal factor [29]. Novice nurse praxis deficits may be attributed to pre-registration education when there is a disconnect between academic expectations, clinical performance and policy [18,23]. Vaismoradi et al. [5] reported that nursing students identified gaps in their education interfered with their ability to provide safe care in the ‘real’ world. Such findings compel educators to address the role of education in the preparation of safe practitioners. Ebright et al. recommended educator reflection to improve teaching and learning patient safety [27]. This need seems particularly relevant given that “[n]ursing students and faculty need to move beyond believing that perfection in performance of nursing procedures, as taught in the nursing skill laboratory, is possible in all actual care situations”[27].

Internationally, literature has mainly focused on defining what constitutes unsafe student practice from the perspective of educators [17]. From this perspective, unsafe clinical students are typically depicted as practitioners with deficits in motivation, psychomotor skills, knowledge, and interpersonal skills. A single study examining student perspectives described a lack of accountability, unprofessionalism or disengagement as indicators of safety risk in clinical learning situations [19]. From students’ perspectives, however, the educator also plays a critical role in ensuring patient safety. Premature autonomy, whereby a student is not appropriately guided by their educator, was viewed as unsafe [19]. This study seeks to expand the literature by describing the viewpoints of senior undergraduate nursing students about compromised safety in the clinical learning environment.

## Methods

### Aim

This study is a component of a larger research initiative that involves all levels of students enrolled in a single baccalaureate nursing program. The aim of the parent study is to conceptualize students’ perspective of safety in clinical learning. Appreciating variability in viewpoints across and between years of study, this paper specifically describes fourth year nursing students` views about when it is most unsafe in the clinical setting.

### Design

Q methodology was used to systematically elicit multiple viewpoints about a defined topic of inquiry [35,36]. The challenging nature of managing data within traditional qualitative approaches presents difficulties during data reduction and generally leads to small sample sizes. In traditional quantitative approaches such as surveys or questionnaires individual subjectivities are lost. Q methodology incorporates the strengths of both qualitative and quantitative techniques to measure subjectivity in a systematic way [9]. Subjectivity is measured through factor analysis of how students rank a given set of statements. This methodology is well suited to be used in nursing research to rigorously examine similarities and differences among people’s thoughts, feelings and perceptions [35].

A Q-sample refers to the statements sorted by the participants. For this study, the Q-sample was refined from an earlier concourse, a collection of statements comprised of experiential, theoretical and evidence-informed viewpoints, collated by two of the authors of this study. This parent concourse of 232 statements, was generated through an integrative literature review [17], student focus groups and consultation with content experts [19]. Further refinement of the parent concourse was warranted for evolving conceptual understanding regarding unsafe student practices in the clinical milieu. To strengthen content and face validity of the statements, the refinement process was guided by the recommendations of Akhtar-Danesh et al. [35]. The concourse was reviewed by the research team to ensure clarity of statements and eliminate duplications. The resultant 63 statements were then pilot tested through individual consultation with nursing program stakeholders, including four students and two nurse educators. Based on the findings from the pilot, the concourse was further refined to incorporate recommendations for ease of completion, simplicity of language and clinical relevancy. As a result 20 statements were eliminated. The final 43 Q-sample statements about student unsafe clinical practices is presented in Table 1.

**Table 1:** Q-sample statements

**Number**	**Statement**
1	The student practices outside of his/her scope
2	The student makes independent clinical decisions beyond his/her competency
3	The student does not consider the guidance of the clinical educator
4	The student does not demonstrate critical thinking through the nursing process
5	The student lacks the knowledge needed to assume care of assigned patients
6	The student is dishonest (makes up assessment data, covers up mistakes or hides their lack of knowledge)
7	The student does not report changing patient conditions
8	The student fails to perform care consistent with clinical guidelines and standard procedures (hand washing; confidentiality; body mechanics)
9	The student rushes through care
10	The student does not respect the rights of patients
11	The student demonstrates a pattern of errors (e.g. repeated mistakes)
12	The student is unable to control his/her nervousness
13	The student does not provide accurate, relevant and timely documentation of client care
14	The student has difficulty communicating (verbally or non-verbally
15	The student avoids interacting with the patient
16	The student avoids consultation and collaboration with the clinical educator and other health team members
17	The student is unable to modify care based on emerging patient priorities
18	The student does not protect patients from injury or potentially abusive situations
19	The student practices with impaired cognition (due to stress, drugs, alcohol, or lack of sleep)
20	The student does not demonstrate patient-centeredness (e.g. caring)
21	The clinical educator does not appropriately guide student practice
22	The clinical educator demonstrates lack of competence in his/her role
23	The student does not have access to ongoing performance feedback
24	The student is evaluated as successful despite a pattern of unmet clinical expectations
25	The student feels overwhelmed by course requirements
26	The student has a large gap in time between practice placements
27	The clinical educator encourages students to do things beyond their scope
28	The clinical educator does not assign clinical learning experiences consistent with student’s learning needs
29	The clinical educator has not established a trusting relationship with the student(s)
30	Clinical educators and professors do not regularly discuss student progress and expectations
31	The clinical educator does not enforce clinical program policies
32	The clinical educator does not regularly document evaluations of the student’s performance
33	There are no clear guidelines for how to deal with specific behaviours in the clinical setting (when to fail someone or what is considered unsafe student practices)
34	There are inconsistent expectations among clinical groups or faculty (skill, workload, hours)
35	The clinical educator is overwhelmed by role expectations
36	The clinical educator is unable to establish and maintain a positive learning environment
37	The formal evaluation processes are unclear
38	The clinical educator does not role model established nursing standards
39	The student responds defensively to constructive feedback
40	The student is taught to cut corners or guided to do things differently than what was taught in school
41	The clinical educator does not provide constructive feedback in a confidential manner
42	The clinical educator does not set clear expectations with students at the beginning or and throughout the placement
43	The student perceives the clinical educator as threatening

### Setting and sample

This study’s setting was a single baccalaureate nursing program with multiple collaborative university and college delivery sites in Northeastern Ontario, Canada. The sites shared a common humanistic educative curriculum. Academic and clinical components were integrated throughout the four year program, culminating in a praxis consolidation during the final year of study. Inclusion criteria were students currently registered in their final year prior to degree completion at one of two sites within the collaboration, subsequently referred to as Site A and Site B. There were no exclusion criteria. A total of 59 participants were recruited through an in-class activity.

### Data collection

**Figure 1:**
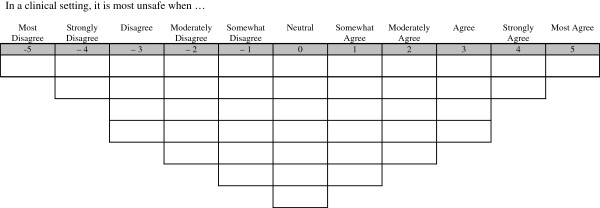
**Q-template. **In a clinical setting, it is most unsafe when.

During an in-class learning activity, students were provided with a package containing a set of 43 individual cards, a Q-template (Figure 1), a two-item demographic profile, a consent form and a pencil. Each double-sided card contained a single Q-sample statement with its assigned number recorded on the reverse side. The Q-template contained 43 spaces arranged as a pyramid with two endpoints labelled as *Most Disagree (−5) and Most Agree (+5).* This range was selected to accommodate the number of statements. It has been suggested that the range of rating scale at the top of the pyramid is wider when there is a large number of statements [35]. In response to the following prompt “In a clinical setting, it is most unsafe when ..,” students placed each statement card into one of the 43 boxes. Once all cards were placed, each participant transferred the number recorded on the back of the statement card onto the corresponding box on the Q-template. The ranking assigned to each statement was designated by the rating identified at the top of the column on the provided Q-template. For example, a statement card placed in the extreme right hand column, *Most Agree*, received a ranking of +5.

### Ethical considerations

Written ethical approval for this study was obtained from the Research Ethics Committee at one of the educational sites. In response, an affirmative written approval to proceed with the study was received from the Chair of the Research Ethics Committee at the remaining partner site. Since students in a classroom may be considered a captive and therefore vulnerable population, a number of considerations were undertaken to preserve anonymity. A complete demographic profile (beyond year of study and previous education) was not collected to ensure anonymity. Participants were informed that their participation or non-participation would not impact on their academic status in the course or the program. The design of this study provided for a study sample from a large group of students during the in-class learning activity. This approach ensured greater anonymity for willing participants and confidentiality of students’ decisions not to participate. To maintain anonymity, participants were instructed to not include any personal identification on the Q-sorts. At the end of the activity the Q-sorts, demographic profiles and completed consent forms were collected. Students who choose not to participate in the study had the option of retaining their Q-sort or handing it in without a signed consent, whereby it was excluded from further analysis.

### Data analysis

The demographic data were analyzed using descriptive statistics. Q-analysis using PQ Method 2.11[37], involved centroid factor analysis and varimax rotation to identify shared viewpoints specific to Site A and Site B. A combined analysis of the data was completed [38]. Analysis of large data sets within Q-methodology, however, may lead to overlooked nuances in the data. Using the guidelines suggested by Brown [36], “What is of interest ultimately are the factors with at least four or five persons defining each; beyond that, additional subjects add very little.” Therefore, analysis of the data from a site-specific level enables the identification of additional meaningful student viewpoints that may have been influenced by contextual factors.

Viewpoints shared by participants within a site were identified through automatic flagging of sorts which loaded as a single discrete factor. The analysis yielded multiple factor arrays. For each site, the factor array selected was comprised of three discrete viewpoints as well as points of consensus. The decision making criteria for this selection was inclusivity of participants and pragmatic utility in accordance with the research question. Initial labelling of factors occurred with students at Site A working in groups of four to six. Students were instructed to consider labels based on statement patterns, while paying particular attention to distinguishing features that were rated highly positive or highly negative [39]. Following small group work, a large group discussion occurred to assist the researchers’ understanding of participants' underlying beliefs and values, which enhanced the validity of results [40]. Through researcher consensus, each site-specific factor was assigned a descriptive label derived from the interpretation of what is unsafe in the clinical setting, as opposed to what is safe.

## Results

In total, 59 fourth year students, (Site A, n = 36; Site B, n = 23) consented to further analysis of their submitted Q-sort. Three quarters of Site A participants and one-third of Site B participants completed some form of post-secondary education prior to entry into the nursing program. The proportion of participants entering into the nursing program immediately following completion of secondary education was 11% for Site A and 56% for Site B. In response to the statement “In a clinical setting, it is most unsafe when …,” three discrete viewpoints and a consensus perspective for each of the two sites are described.

### Site A discrete viewpoint 1: endorsement of uncritical knowledge transfer

This first viewpoint, labelled, *Endorsement of Uncritical Knowledge Transfer*, is composed of 17 distinguishing statements (Table 2). The 13 Site A students who share this perspective perceived that it was most unsafe when limited application of knowledge jeopardized patient-centered praxis. This situation occurs when students are taught to cut corners (40/+3 [statement number/ranking]), are unable to modify care based on changing patient needs (17/+2), receive successful evaluations despite unsuccessful clinical performance (24/+2), and fail to communicate essential patient information (7/+2). In addition, critical knowledge transfer is jeopardized when professional role models are absent (38/+2). Three neutral statements describe three barriers to knowledge transfer including a lack of preparatory knowledge (5/0), violation of established guidelines (8/0) and a lack of processes to address deficits and minimize risk (33/0). Statements least representative of this viewpoint relate to program features. This includes the structure of clinical placements (26/-5), students’ reaction to program expectations (25/-4) and student/educator relationships (41/-3).

**Table 2:** Site A: Endorsement of uncritical knowledge transfer

**Numbered statement**		**Statement rankings across discrete viewpoints**		
		**1**	**2**	**3**
40	The student is taught to cut corners or guided to do things differently than what was taught in school	**3**	2	1
17	The student is unable to modify care based on emerging patient priorities	**2**	0	1
24	The student is evaluated as successful despite a pattern of unmet clinical expectations	**2**	1	1
7	The student does not report changing patient conditions	**2**	3	3
38	The clinical educator does not role model established nursing standards	**2**	−2	0
31	The clinical educator does not enforce clinical program policies	**1**	−3	−1
5	The student lacks the knowledge needed to assume care of assigned patients	**0**	−1	2
8	The student fails to perform care consistent with clinical guidelines and standard procedures (e.g. hand washing; confidentiality; body mechanics)	**0**	−2	3
33	There are no clear guidelines for how to deal with specific student behaviours in the clinical setting (when to fail someone or what is considered unsafe student practices)	**0**	−4	−2
30	Clinical educators and professors do not regularly discuss student progress and expectations	**−1**	−3	−3
35	The clinical educator is overwhelmed by role expectations	**−1**	−2	−2
36	The clinical educator is unable to establish and maintain a positive learning environment	**−2**	2	0
14	The student has difficulty communicating (verbally or non-verbally)	**−2**	1	−2
29	The clinical educator has not established a trusting relationship with the student(s)	**−2**	0	−1
41	The clinical educator does not provide constructive feedback to students in a confidential manner	**−3**	−1	−1
25	The student feels overwhelmed by course requirements	**−4**	3	−5
26	The student has a large gap in time between practice placements	**−5**	1	−3

### Site A discrete viewpoint 2: Non-student centered program

This second viewpoint of 18 distinguishing statements is entitled, *Non-Student Centered Program* (Table 3)*.* The five Site A students who share this viewpoint, perceive a disconnect between theory and practice. Safety is most at risk when students are overwhelmed by program expectations (25/+3), lack trust in their educator’s competency (22/+3) and ability to facilitate their learning (36/+2; 43/+2). The two neutral statements refer to a lack of regard for patients (10/0) and their needs (17/0). The negatively ranked statements include an absence of boundaries (33/-4), a lack of adherence to program policies (31/-3), and defensive student responses to constructive appraisal (39/-3).

**Table 3:** Site A viewpoint 2: Non-student centered program

**Numbered statements**		**Statement rankings across discrete viewpoints**		
		**1**	**2**	**3**
25	The student feels overwhelmed by course requirements	−4	**3**	−5
22	The clinical educator demonstrates lack of competence in his/her role	1	**3**	2
36	The clinical educator is unable to establish and maintain a positive l earning environment	−2	**2**	0
43	The student perceives the clinical educator as threatening	−1	**2**	−1
26	The student has a large gap in time between practice placements	−5	**1**	−3
14	The student has difficulty communicating (verbally or non-verbally)	−2	**1**	−2
27	The clinical educator encourages students to do things beyond their scope	3	**1**	2
10	The student does not respect the rights of patients	1	**0**	2
17	The student is unable to modify care based on emerging patient priorities	2	**0**	1
5	The student lacks the knowledge needed to assume care of assigned patients	0	**−1**	2
34	There are inconsistent expectations among clinical groups or faculty (skill, workload, hours)	−2	**−1**	−3
12	The student is unable to control his/her nervousness	−4	**−1**	−4
8	The student fails to perform care consistent with clinical guidelines (e.g. hand washing; confidentiality; body mechanic)	0	**−2**	3
38	The clinical educator does not role model established nursing standards	2	**−2**	0
4	The student does not demonstrate critical thinking through the nursing process	0	**−2**	0
31	The clinical educator does not enforce clinical program policies	1	**−3**	−1
39	The student responds defensively to constructive feedback	−1	**−3**	−1
33	There are no clear guidelines for how to deal with specific behaviours in the clinical setting (when to fail someone or what is considered unsafe student practices)	0	**−4**	−2

### Site A discrete viewpoint 3: overt patterns of unsatisfactory clinical performance

This third viewpoint, *Overt Patterns of Unsatisfactory Clinical Performance*, is comprised of 15 distinguishing statements (Table 4). The 12 students who share this viewpoint perceive repeated deficits in knowledge and performance of fundamental clinical procedures as most indicative of unsafe practice (8/+3; 11/+3; 5/+2). Three of the four neutral statements address clinical educator role deficits (38/0; 21/0; 36/0). The least important statements describe students’ response in the learning environment and program structure (25/-5; 26/-3).

**Table 4:** Site A viewpoint 3: Overt patterns of unsatisfactory clinical performance

**Numbered statements**		**Statement rankings across discrete viewpoints**		
		**1**	**2**	**3**
8	The student fails to perform care consistent with clinical guidelines and standard procedures (e.g. hand washing; confidentiality; body mechanics)	0	−2	**3**
11	The student demonstrates a pattern of errors (e.g. repeated mistakes)	2	1	**3**
5	The student lacks the knowledge needed to assume care of assigned patients	0	−1	**2**
17	The student is unable to modify care based on emerging patient priorities	2	0	**1**
16	The student avoids consultation and collaboration with the clinical educator and other health team members	−1	−1	**1**
20	The student does not demonstrate patient-centeredness (e.g. caring)	0	−2	**0**
38	The clinical educator does not role model established nursing standards	2	−2	**0**
21	The clinical educator does not appropriately guide student practice	1	2	**0**
36	The clinical educator is unable to establish and maintain a positive learning environment	−2	2	**0**
31	The clinical educator does not enforce clinical program policies	1	−3	**−1**
28	The clinical educator does not assign clinical learning experiences consistent with student’s learning needs	−3	−3	**−2**
14	The student has difficulty communicating (verbally or non-verbally)	−2	1	**−2**
33	There are no clear guidelines for how to deal with specific student behaviours in the clinical setting (when to fail someone or what is considered unsafe student practices)	0	−4	**−2**
26	The student has a large gap in time between practice placements	−5	1	**−3**
25	The student feels overwhelmed by course requirements	−4	3	**−5**

### Site A consensus viewpoint: contravening practices

There is agreement across Site A students, regardless of their discrete factor loading, on the ranking of 14 statements. This consensus viewpoint is labelled *Contravening Practices* (Table 5). Positively ranked statements focus on student action or inaction incongruent with expectations of the profession. In particular, those statements positively ranked from +5 to +2 address students’ violations of professional boundaries, such as a failure to fulfill one’s role with regards to scope of practice (1), patient protection (18) and integrity (6). Relatively neutral rankings about clinical safety (ranking ranged from +1 to 0) describe rushing through care (9), documentation deficits (13), lack of receptivity to guidance from clinical educators (3) and avoidance of patient interaction (15). The negatively ranked statements (ranging from −1 to −4) predominantly focus on the educators’ role as evaluator (42, 23, 34, 32). Finally, safety was somewhat compromised when clinical educators are perceived to be overwhelmed by their role (35, ranking ranged from −1 to −2).

**Table 5:** Site A consensus viewpoint: Contravening practices

**Numbered statements**		**Statement rankings across discrete viewpoints**		
		**1**	**2**	**3**
1	The student practices outside of his/her scope	4	4	5
6	The student is dishonest (makes up assessment data, covers up mistakes or hides their lack of knowledge)	3	4	4
2	The student makes independent clinical decisions beyond his/her practice	4	3	2
18	The student does not protect patients from injury or potentially abusive situations	3	2	4
9	The student rushes through care	1	1	1
13	The student does not provide accurate, relevant and timely documentation of client care	0	0	1
3	The student does not consider the guidance of the clinical educator	1	0	0
15	The student avoids interacting with the patient	0	0	0
20	The student does not demonstrate patient-centeredness (e.g. caring)	0	−2	0
42	The clinical educator does not set clear expectations with students at the beginning of and throughout the placement	−1	0	−1
35	The clinical educator is overwhelmed by role expectations	−1	−2	−2
23	The student does not have access to ongoing performance feedback	−2	−1	−3
34	There are inconsistent expectations among clinical groups or faculty (skill, workload, hours)	−2	−1	−3
32	The clinical educator does not regularly document evaluations of the student’s performance	−3	−4	−2

### Site B discrete viewpoint 1: premature and inappropriate clinical progression

The Site B viewpoint, labelled, *Premature and Inappropriate Clinical Progression*, is composed of 7 distinguishing statements (Table 6). The 11 students who share this perspective perceive that safety is most compromised when students make decisions beyond their capacity (2/+4), at times with the encouragement of their clinical educator (27/+3), and reinforced through the granting of academic success despite a pattern of unmet clinical expectations (24/+1; 13/+1). There are no neutral statements in this viewpoint. The least representative statement describes students’ inability to mask their nervousness (12/-4). A remaining negatively ranked statement addresses the educator’s inability to ensure a positive clinical learning environment (36/-2). A perceived lack of dialogue between educators and professors also compromises clinical expectations and outcomes (30/-1).

**Table 6:** Site B viewpoint 1: Premature and inappropriate clinical progression

**Numbered statements**		**Statement rankings across discrete viewpoints**		
		**1**	**2**	**3**
2	The student makes independent clinical decisions beyond his/her competency	**4**	−1	2
27	The clinical educator encourages students to do things beyond their scope	**3**	2	−3
24	The student is evaluated as successful despite a pattern of unmet clinical expectations	**1**	2	2
13	The student does not provide accurate, relevant and timely documentations of client care	**1**	2	−1
30	Clinical educators and professors do not regularly discuss student progress and expectations	**−1**	−3	−5
36	The clinical educator is unable to establish and maintain a positive learning environment	**−2**	0	0
12	The student is unable to control his/her nervousness	**−4**	−2	−1

### Site B discrete viewpoint 2: Non-patient centered practice

This second viewpoint, labeled *Non-Patient Centered Practice* is composed of 14 distinguishing statements (Table 7). This represents the view of 6 site B students. It is characterized by a failure of educators to adhere to practice boundaries (27/+2) and enforce program policies (31/+1). Within this context, students do not provide professional documentation of patient care (13/+2) and ultimately fail to protect patients from risk (18/+4). The single neutral statement describes practice inconsistent with academic knowledge (40/0). The four statements ranked as the least relevant to patient-centered practices focus on ineffective teaching and learning processes (39/-5; 34/-4; 41/-4; 30/-3).

**Table 7:** Site B viewpoint 2: Non-patient centered practice

**Numbered statements**		**Statement rankings across discrete viewpoints**		
		**1**	**2**	**3**
18	The student does not protect patients from injury or potentially abusive situations	1	**4**	2
27	The clinical educator encourages students to do things beyond their scope	3	**2**	−3
13	The student does not provide accurate, relevant and timely documentation of client care	1	**2**	−1
31	The clinical educator does not enforce clinical program policies	−1	**1**	−2
40	The student is taught to cut corners or guided to do things differently than what was taught in school	2	**0**	1
2	The student makes independent clinical decisions beyond his/her competency	4	**−1**	2
1	The student practices outside of his/her scope	5	**−1**	5
32	The clinical educator does not regularly document evaluations of the student’s performance	−4	**−1**	−3
4	The student does not demonstrate critical thinking through the nursing process	1	**−2**	1
37	The formal evaluation processes are unclear	−5	**−2**	−4
30	Clinical educators and professors do not regularly discuss student progress and expectations	−1	**−3**	−5
41	The clinical educator does not provide constructive feedback in a confidential manner	−2	**−4**	−1
34	There are inconsistent expectations among clinical groups or faculty (skill, workload, hours)	−1	**−4**	0
39	The student responds defensively to constructive feedback	−2	**−5**	−3

### Site B discrete viewpoint 3: negating purposeful interactions for experiential learning

The third viewpoint, titled *Negating Purposeful Interactions for Experiential Learning* is composed of 10 distinguishing statements (Table 8). This perspective, shared by 4 students, identifies impaired relations between and among students, clinical educators and patients (22/+3; 20/+2; 2/+2). Within this viewpoint, the perceived lack of a clinical educator’s role competence is identified as the most indicative of high risk for unsafe practice. The three neutral statements embody the inability of students to engage in humanistic caring (10/0; 17/0; 25/0). Academic dialogue between educators and program faculty (30/-5) as well as a lack of program resources (33/-4) are of least importance for experiential learning.

**Table 8:** Site B viewpoint 3: Negating purposeful interactions for experiential learning

**Numbered statements**		**Statement rankings across discrete viewpoints**		
		**1**	**2**	**3**
22	The clinical educator demonstrates lack of competence in his/her role	1	1	**3**
20	The student does not demonstrate patient-centeredness	−3	−3	**2**
2	The student makes independent clinical decisions beyond his/her competency	4	−1	**2**
10	The student does not respect the rights of patients	2	2	**0**
17	The student is unable to modify care based on emerging patient priorities	3	3	**0**
25	The student feels overwhelmed by course requirements	−3	−3	**0**
13	The student does not provide accurate, relevant and timely documentation of client care	1	2	**−1**
27	The clinical educator encourages students to do things beyond their scope	3	2	**−3**
33	There are no clear guidelines for how to deal with specific behaviours in the clinical setting (when to fail someone or what is considered unsafe student practices)	0	0	**−4**
30	Clinical educators and professors do not regularly discuss student progress and expectations	−1	−3	**−5**

### Site B consensus viewpoint: eroding conventions

A shared perspective for students at site B identifies student dishonesty (6), a lack of knowledge (5), and unsatisfactory performance (11, 7, 8, 9) as most indicative of *Eroding Conventions* (Table 9). The shared rankings of these statements (ranging from +4 to +1) represent a threat to the fundamentals of nursing by senior nursing students. Students at greatest risk of effacing accepted professional protocols did not engage in authentic dialogue with clinical educators and others (24, 16, 21, 3, 15; rankings ranged from +2 to 0). Clinical educator’s role performance (38, 35) is moderately influential (ranging in ranking from 0 to −2) in this perspective. Those statements having least impact on upholding established conventions (ranging in ranking from 0 to −4) are deficits in program structure (26) and student interactions with educators (12, 43, 42, 23).

**Table 9:** Site B consensus viewpoint: Eroding conventions

**Numbered statements**		**Statement rankings across discrete viewpoints**		
		**1**	**2**	**3**
6	The student is dishonest (makes up assessment data, covers up mistakes or hides their lack of knowledge)	4	4	3
5	The student lacks the knowledge needed to assume care of assigned patients	2	3	4
11	The student demonstrates a pattern of errors (e.g. repeated mistakes)	2	3	3
7	The student does not report changing patient conditions	3	3	2
8	The student fails to perform care consistent with clinical guidelines and standard procedures (hand washing; confidentiality; body mechanics)	2	2	3
24	The student is evaluated as successful despite a pattern of unmet clinical expectations	1	2	2
9	The student rushes through care	1	1	1
16	The student avoids consultation and collaboration with the clinical educator and other health team members	0	1	1
21	The clinical educator does not appropriately guide student practice	0	1	0
3	The student does not consider the guidance of the clinical educator	0	1	0
14	The student has difficulty communicating (verbally or non-verbally)	0	0	1
15	The student avoids interacting with the patient	0	0	1
38	The clinical educator does not role model established nursing standards	−1	0	−2
23	The student does not have access to ongoing performance feedback	−1	−1	−2
35	The clinical educator is overwhelmed by role expectations	−2	−1	−2
42	The clinical educator does not set clear expectations with students at the beginning or and throughout the placement	−1	−2	−2
43	The student perceives the clinical educator as threatening	−3	−1	−1
12	The student is unable to control his/her nervousness	−4	−2	−1
26	The student has a large gap in time between practice placements	−3	−3	−3

## Discussion

This study described senior undergraduate nursing students’ emic understanding of unsafe clinical practices and learning contexts. Across both sites in this study, the resultant six viewpoints and two consensus perspectives support the premise that by virtue of their humanness, senior students are aware of their fallibility. As such, students are not immune to involvement in practices that threaten patient safety. The senior students’ perspectives of when it is most unsafe in the clinical setting reveal concerns that warrant consideration for strengthening nursing curricula to render safety praxis overt. Educators mindful of multiple students’ perspectives about clinical safety have the potential to promote professional integrity through shared consciousness and supportive educative learning partnerships [1,11,26,41].

Unique to Site A is a single discrete viewpoint that emphasizes the student’s accountability for safety. This perspective, *Overt Patterns of Unsatisfactory Performance,* identifies that students are most unsafe if they are unprepared, lack knowledge and engage in a pattern of errors. The focus of clinical safety for these students, regardless of the quality and quantity of systemic supports, is individual competence. This viewpoint supports the need for concurrent integration of safety knowledge, skills and attitudes with clinical learning to ensure individual competence [5,30,32]. Without such development, it is most unsafe in the clinical setting. This perspective is similar to a blame-oriented understanding of error causation by individual practitioners [16]. Sole responsibility for clinical safety is problematic however, given that central role of educators in the development and evaluation of student competence. Skilled educators balance the student’s right to learn in the clinical setting with the patient’s right to competent care. In the presence of *Overt Patterns of Unsatisfactory Clinical Performance* the patient’s right to competence care must supersede the student’s right to learn in the clinical setting. In such circumstances, immediate student removal from the clinical setting is imperative. To foster the student’s potential as a safe novice practitioner and uphold the patient’s right to safety, professional development initiatives for educators regarding their roles and responsibilities is fundamental to supporting a contemporary curriculum aligned with the safety mandate.

System accountability for patient safety is most evident across the remaining discrete viewpoints for Site A and Site B participants. These viewpoints suggest that students’ predominantly understood safety as a shared rather than individual responsibility [20,42]. More specifically, the responsibility to uphold a culture of safety is not only the purview of students, but also educators and the nursing program as a whole. These findings support the importance of creating clinical learning environments constituted by structures, processes and practices that align with safety. It is important to note that a culture of safety does not negate an individual’s accountability for safeguarding patients [12,19,33]. Rather, it emphasizes the importance of partnerships focused on continuous quality improvement [15]. As partners in the learning process, educators are responsible for guiding and evaluating students’ development of entry level competencies to uphold safety standards. Students, also members of the learning partnership, are responsible for their individual development according to professional and program standards.

All those involved in the teaching and learning of nursing must be vigilant for error prevention, detection, reporting, analysis, and if warranted, individual remediation and system reconfiguration. The findings at both program sites support the premise that vigilance is not limited to attentiveness for clinical errors, but is the impetus for the creation of a culture of safety [27]. The results also expand this notion to shared vigilance as the simultaneous, conscious and sustained expenditure of effort to knowledgeably attend to program standards, procedures, regulatory guidelines, practice boundaries in variable care contexts by both educators and students.

A competency that is foundational to the adherence of standards, procedures, guidelines, and boundaries is critical thinking in practice [14,30]. From a student perspective, effective teaching and learning approaches aimed at promoting clinical judgment include conceptual mapping, case studies and collaborative decision making for patient care [43]. Each of these strategies is strengthened with regular authentic dialogue between students and educators for the purpose of articulating “what they know, how they know it, and who they are in nursing” (p. 135) [43]. Collectively, the discrete student perspectives in this study acknowledge the merit of student educator interactions for safe experiential learning.

The importance of open dialogue about patient safety and clinical errors is not unique to nursing. Within medical education, Halbach and Sullivan’s [44] work supports the success of brief, forthright discussions between faculty and intermediate medical students about patient safety issues. Although these authors acknowledge a dearth of evidence for incorporating specifics about safe practice into the education of health professions, they advocate for the use of active experiential learning strategies. These include, but are not limited to genuine one-to-one discussions between students and educators, role-play, simulations, and small group peer discussions. Similarly, in nursing, the use of strategic interactions between students and educators offer an opportunity to increase knowledge about patient safety and in turn, prevent clinical errors [25,26,30,41].

The two consensus perspectives, *Contravening Practices* (Site A) and *Eroding Conventions* (Site B) implore both educators and students to demonstrate nursing ontological, epistemological and praxis fundamentals to minimize safety risks. Two statements that were similarly ranked as positive indicators of unsafe clinical practice at both Site A and B were dishonesty and rushing through care. These two points of agreement suggest that professional morality and patient-centered care are areas that warrant emphasis in safety curricula. Further, positively ranked statements suggest that safety is threatened when established expectations for sound clinical judgement and action are violated by students. Based on these findings, it is imperative that nursing curricula explicitly detail the cognitive, moral, and practice parameters of safe practice to engender safety among students in the clinical setting. Unique to the consensus perspective at Site B is the importance assigned to clinical educators’ role in preserving safety through student evaluations. The rigor of clinical evaluations are influenced by educator role confidence and competence, and clarity of program expectations and policies [17,41]. These findings support the need for ongoing development of educators as competent safety ambassadors [30,32,45]. Overall, senior nursing students agree that the absence of a moral consciousness, patient-centeredness, and professional competency renders clinical safety violations inevitable.

A limitation of this study was time intensive nature of the Q-sorting process by students. Some students requested additional time to thoughtfully sort the 43 Q-sample statements. The findings of this study are not generalizable, nor is this an aim of Q-methodology. The results however, offer a conceptual representation of potential areas for thoughtful consideration by educators in the mitigation of unsafe clinical practices in their respective curricula.

## Conclusions

This study allowed for identification of shared subjectivities from the perspective of students about safety in the clinical setting - a group not typically accessed in the creation of evidence in this area of interest. Students' perceptions of unsafe practice align with a call for system attention to patient safety, a recognized principle of a high-performing health care system. Appreciating that context shapes experiences and perspectives, members of the broader international nursing research, education and practice sectors are encouraged to build upon these results by carrying out comparable methods within their own contexts. In the interim, it is suggested that stakeholders, inclusive of students, attend to their independent and interdependent responsibilities relative to safety. This study’s results may guide intersectoral dialogue on topics such as deficits in knowledge, patient-centered practice, professional morality and authenticity. Such dialogue has the potential to invoke new or renewed cooperative strategies for safety within unique and complex multi-dimensional contexts of learning and practice. Overall, in an effort to eradicate compromised safety associated with clinical learning, stakeholders must cooperatively embody the ontological, epistemological and praxis fundamental of nursing.

## Competing interests

There are no competing interests.

## Authors’ contributions

All authors participated in the conception and/or conduction of the study. Each author was involved in preparing this manuscript. All authors read and approved the final manuscript.

## Funding

This was a non-funded study.

## Pre-publication history

The pre-publication history for this paper can be accessed here:

http://www.biomedcentral.com/1472-6955/11/26/prepub
